# Colloidal Quantum Dot Bulk Heterojunction Solids with Near‐Unity Charge Extraction Efficiency

**DOI:** 10.1002/advs.202000894

**Published:** 2020-06-17

**Authors:** Min‐Jae Choi, Se‐Woong Baek, Seungjin Lee, Margherita Biondi, Chao Zheng, Petar Todorovic, Peicheng Li, Sjoerd Hoogland, Zheng‐Hong Lu, F. Pelayo García de Arquer, Edward H. Sargent

**Affiliations:** ^1^ Department of Electrical and Computer Engineering University of Toronto 10 King's College Road Toronto ON M5S 3G4 Canada; ^2^ Department of Material Science and Engineering University of Toronto 184 College St Toronto ON M5S 3E4 Canada; ^3^ Present address: Department of Chemical and Biological Engineering Korea University 145 Anam‐Ro Seongbuk‐Gu Seoul 02841 South Korea

**Keywords:** bulk heterojunctions, colloidal quantum dots, doping, infrared optoelectronics, light harvesting

## Abstract

Colloidal quantum dots (CQDs) are of interest for optoelectronic applications owing to their tunable properties and ease of processing. Large‐diameter CQDs offer optical response in the infrared (IR), beyond the bandgap of c‐Si and perovskites. The absorption coefficient of IR CQDs (≈10^4^ cm^−1^) entails the need for micrometer‐thick films to maximize the absorption of IR light. This exceeds the thickness compatible with the efficient extraction of photogenerated carriers, a fact that limits device performance. Here, CQD bulk heterojunction solids are demonstrated that, with extended carrier transport length, enable efficient IR light harvesting. An in‐solution doping strategy for large‐diameter CQDs is devised that addresses the complex interplay between (100) facets and doping agents, enabling to control CQD doping, energetic configuration, and size homogeneity. The hetero‐offset between *n*‐type CQDs and *p*‐type CQDs is manipulated to drive the transfer of electrons and holes into distinct carrier extraction pathways. This enables to form active layers exceeding thicknesses of 700 nm without compromising open‐circuit voltage and fill factor. As a result, >90% charge extraction efficiency across the ultraviolet to IR range (350–1400 nm) is documented.

## Graphical abstract

Colloidal quantum dot bulk heterojunction solids for efficient infrared solar cells are presented. A mixture of *n*‐type CQDs and *p*‐type CQDs enables a photoelectric conversion efficiency of >80% in the spectral regime beyond silicon's bandgap and lead to a 17% PCE relative improvement compared to that of best previous reported CQD solids.

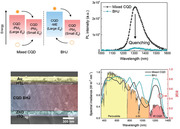

Colloidal quantum dots (CQDs) are promising semiconductor materials for optoelectronic applications owing to their tunable optical properties,^[^ ^1^, ^2^ ^]^ ambient stability,^[^ ^3^, ^4^ ^]^ and room‐temperature solution processing.^[^ ^5^, ^6^ ^]^ The broad bandgap tunability of CQDs allows their absorption and emission to be extended well into the infrared (IR), a region of interest in optical communications,^[^ ^7^ ^]^ night vision,^[^ ^8^ ^]^ biological imaging,^[^ ^9^ ^]^ and IR photovoltaics.^[^ ^10^, ^11^, ^12^ ^]^

The optical response of lead sulfide (PbS) CQDs can be tuned in the range of 980–1750 nm by changing their diameters from 3.8 to 8.0 nm.^[^ ^13^, ^14^ ^]^ Large‐diameter PbS CQDs absorb IR light beyond the response of perovskite (*E*_g_ = 1.58–1.68 eV) and c‐Si (*E*_g_ = 1.12 eV) materials. Thanks to this IR bandgap, IR CQD solar cells have been recently explored as a platform to complement other photovoltaic technologies (e.g., perovskite solar cells, c‐Si solar cells), since they add extra power conversion efficiency (PCE) in a four‐terminal tandem configuration.^[^ ^15^, ^16^, ^17^ ^]^

Unfortunately, the short‐circuit current density (*J*_sc_) of CQD solar cells in the IR solar spectrum (*E*_g_ < 1.12 eV) has continued to reside significantly below its potential. This originates from the fact that a low diffusion length of large‐diameter CQD solids (≈100 nm)^[^ ^18^ ^]^ limits the optimal thickness of IR CQD solar cells (300–500 nm).^[^ ^16^, ^19^ ^]^ The absorption coefficient of IR CQD solids at the first exciton peak is ≈10^4^ cm^−1^,^[^ ^19^ ^]^ thus micrometer‐thick films are required to fully absorb the IR light.

One strategy to increase transport length is to build a CQD bulk heterojunction (BHJ) architecture. This provides separate physical paths for electrons and holes, enabling efficient carrier transport and longer carrier lifetime.^[^ ^20^, ^21^, ^22^ ^]^ To achieve IR CQD BHJ solids, two different types (*n* type and *p* type) of IR CQD inks—dispersions of quantum dots that can be directly used to make a device without further processing (e.g., ligand exchanges)—are needed, and this requires colloid‐phase doping.

We recently reported a cascade surface modification strategy that enables simultaneous control over doping and solubility in CQD inks.^[^ ^23^ ^]^ We achieved doping of small‐diameter CQD inks (*E*_g_ = 1.3 eV) by reprogramming using functional ligands. In large‐diameter CQDs, however, more (100) facets are exposed on the surface whereas small‐diameter CQD mostly consisting of (111) facets^[^ ^14^ ^]^: the different facet arrangement of large‐diameter CQDs (*E*_g_ < 1.1 eV) results in failure of the surface modification strategy that was used for small‐diameter CQDs.^[^ ^24^ ^]^

Here we report a colloid‐phase doping strategy for large‐diameter CQDs that enables the synthesis of IR CQD inks to program their doping type and, ultimately, their band offsets, while retaining size homogeneity. We accomplish this by the use of doping ligands that do not interact with the (100) facets at the CQD surface; whereas prior approaches developed for small‐diameter CQDs lead to CQD fusion when transposed directly to large‐diameter CQDs. We then showcase the IR CQD BHJ solids by using *n*‐type CQDs and *p*‐type CQDs, targeting IR solar cell applications. The IR CQD BHJ structure provides an increased diffusion length, allowing the use of a 700‐nm‐thick active layer in a device that exhibits broadband near‐unity charge extraction efficiency in the range of 350–1400 nm. As a consequence, the external quantum efficiency (EQE) of devices reaches over 80% at the excitonic peak of both *p*‐type (1180 nm) and *n*‐type (1250 nm) large‐diameter CQDs. As a result, the CQD BHJ device reaches a *J*_sc_ of 37 mA cm^−2^ under AM1.5 solar illumination, which corresponds to 17 mA cm^−2^ beyond perovskite (*E*_g_ = 1.61 eV) and 5.5 mA cm^−2^ beyond c‐Si (*E*_g_ = 1.1 eV).

We began by considering the distribution of IR light in the AM1.5 solar spectrum of interest to IR solar cells. The IR light is spectrally distributed, while the absorption of CQD films is concentrated at the first excitonic peak (**Figure** **1**a). We thus employed a multi‐*E*_g_ CQD ensemble (i.e., same surface ligand but different *E*_g_) to match the absorption spectrum of CQD solids with the IR light spectrum.^[^ ^25^ ^]^ Multi‐*E*_g_ CQD inks that are passivated with lead iodide (PbI_2_)—a recently published high‐performing surface passivation^[^ ^5^, ^24^ ^]^—were mixed in solution and cast to form mixed CQD solids. These CQD solids were used to fabricate IR CQD solar cells which consist of ITO/ZnO/mixed CQD/PbS–1,2‐ethanedithiol (EDT)/Au, where ZnO layer is the electron transport layer, mixed CQD layer is the IR light harvesting layer, and PbS–EDT layer acts as a hole transport layer (Figure 1b). Figure 1c displays the absorption spectra of devices using CQD mixture (1:1 ratio) of large *E*_g_ CQDs (*E*_g_ = 1180 nm) and small *E*_g_ CQDs (*E*_g_ = 1250 nm). The mixed CQD solids enable to extend absorption spectra in the IR light spectrum. While ≈700‐nm‐thick CQD solids are required to absorb most of the IR light (>80%), IR device performance—measured by *J*_sc_ × fill factor (FF) with solar spectrum longer than 1100 nm—decreases when the active layer is thicker than 515 nm (Figure 1d). This suggests that the extraction length is limited to ≈500 nm when one employs the previously reported strategy.

**Figure 1:**
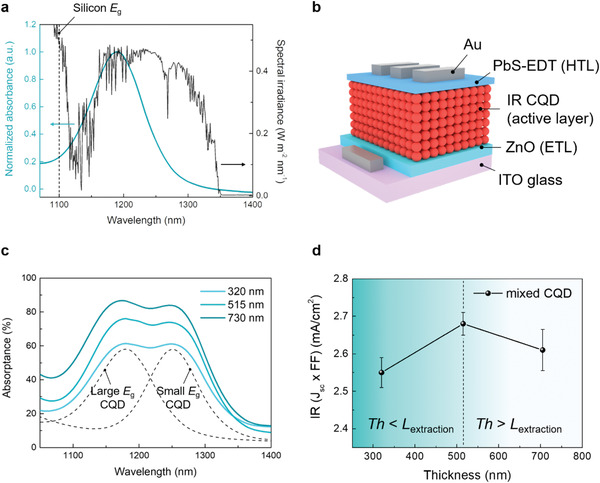
Multi‐*E*_g_ CQDs to maximize IR light harvesting and their limitation. a) Normalized absorbance of a CQD film on glass substrate and spectral irradiance of AM1.5 solar spectrum beyond response of silicon. b) Schematic image of IR CQD solar cells. c) Absorbance of the CQD films with mixture of large *E*_g_ CQDs (*E*_g_ = 1180 nm) and small *E*_g_ CQDs (*E*_g_ = 1250 nm). Mixed ratio of CQDs is 1:1. Dashed lines indicate absorption spectra of large *E*_g_ CQDs and small *E*_g_ CQDs, respectively. d) Thickness‐dependent *J*_sc_ × FF of IR CQD solar cells with solar light beyond 1100 nm wavelength. Mixed CQDs are used as active layer for devices. The performance decreases when the active layer is thicker than 515 nm. *Th* is the thickness of CQD solid and *L*_exctration_ is the extraction length of carrier in the CQD solid.

We therefore pursued IR CQD BHJ solids—a mixture of multi‐*E*_g_ CQD with different doping types—to enhance carrier transport by providing distinct physical paths for each type of carrier. Since PbI_2_‐passivated CQDs exhibit *n*‐type doping character,^[^ ^26^, ^27^ ^]^ a doping strategy is required to render the films *p* type. We reprogrammed the surface of PbI_2_‐passivated large‐diameter CQD inks (CQD‐PbI_2_) with functional ligands to provide a *p*‐type character.^[^ ^23^, ^26^, ^28^ ^]^ During our experiments on doping of inks of small‐diameter CQDs, we found that surface reprogramming with cysteamine (CTA) ligand enables the best colloidal solubility.^[^ ^23^ ^]^ We therefore extended this approach to large‐diameter CQD inks. In brief, diluted CTA solution was slowly introduced into PbI_2_‐capped IR CQD inks during stirring (**Figure** **2**a). The degree of doping was tuned by controlling the amount of CTA added to the CQD inks. The atomic ratio of CQD inks changed after CTA reprogramming, as evidenced by X‐ray photoelectron spectroscopy (XPS) measurements (Table S1, Supporting Information): an increase of sulfur to lead and a decrease of iodine to lead indicate a replacement of PbI_2_ with CTA at the CQD surface.

**Figure 2:**
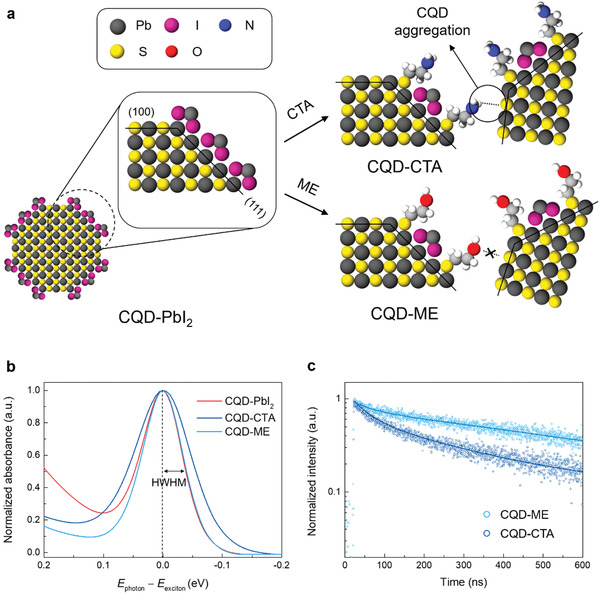
Colloid‐phase doping for large‐diameter CQD inks. a) Ligand doping process through surface reprogramming of CQD inks. The CQD inks passivated with PbI_2_ (*n* type) were treated with CTA and ME doping ligands to form *p*‐type CQD inks (CQD‐CTA and CQD‐ME). For CQD CTA inks, aggregation of CQDs occurs due to the attachment of amine functional group to (100) facets. b) Normalized absorption spectra of the CQD solids after ligand exchange. c) TRPL measurement of CQD inks. The signal is fit using a biexponential function to calculate carrier lifetime (solid line).

CTA‐reprogramming (CQD‐CTA) results in a 1.3× increase of half‐width at half‐maximum (HWHM) of large‐diameter CQDs from 39 to 55 meV (Figure 2b): additional polydispersity is produced^[^ ^2^, ^5^ ^]^ by the CTA reprogramming, which is not shown in the case of small‐diameter CQDs.^[^ ^23^ ^]^ Large‐diameter CQDs have unpassivated (100) facets at their surface,^[^ ^14^, ^24^ ^]^ which can be passivated by the amine functional group (—NH_2_) in CTA.^[^ ^29^, ^30^ ^]^ Therefore, CTA ligands induce aggregation of CQDs in solution: the thiol functional group (—SH) binds to (111) facet of one dot and —NH_2_ binds to (100) facet of another dot (Figure 2a). This aggregation can lead to heterogeneous CQD fusion during the process,^[^ ^31^ ^]^ and this can produce an increase of energetic disorder.

To assess this hypothesis, we employed density functional theory (DFT) to investigate CTA passivation of (100) PbS surfaces (Figure S1 Supporting Information). Calculations reveal that the most stable configuration is that in which —NH_2_ binds to Pb atoms from (100) PbS surfaces and —SH points outward. This agrees with the idea that —SH groups may contribute to aggregation of CQDs, such as by binding to (111) facets of other CQDs.

We therefore turned our attention to doping ligands that would retain the homogeneity of the CQD inks. We used mercaptoethanol (ME)—a functional ligand consisting of one hydroxyl group (—OH) and one thiol group—reasoning that the hydroxyl group does not attach to the (100) facets of PbS CQDs.^[^ ^32^ ^]^ To reprogram the CQD surface with ME, we introduced diluted ME solution in dimethylformamide (DMF) into PbI_2_‐passivated IR CQD inks. This led to a decrease of the I 3d signal, while a thiol signal in the S 2p spectra appears (Figure S2, Supporting Information). The atomic ratio of iodine to lead decreases from 0.62 to 0.40, while the sulfur to lead ratio increases from 0.88 to 1.27.

The ME‐reprogrammed IR CQD inks (CQD‐ME) retain their HWHM, which indicates minimization of polydispersity (Figure 2b). We further carried out time‐resolved photoluminescence measurements of CQD inks in order to investigate the effect of polydispersity on carrier recombination (Figure 2c). The CQD‐ME inks exhibit 2.3× longer carrier lifetime (488 ns) compared to the CQD‐CTA inks (208 ns). These photophysical studies suggest that facet arrangement of large‐diameter CQDs results in different homogeneity of CQD inks depending on doping ligand.

To study the doping properties of the CQD inks, we measured ultraviolet photoelectron spectroscopy (UPS). **Figure** **3**a,b show the UPS spectra of the high‐binding‐energy cutoff (Fermi level) and low‐binding‐energy cutoff (valence band), respectively. The energy difference between the Fermi level (*E*_F_) and the valence band (VB) decreases from 0.57 eV (CQD‐PbI_2_) to 0.45 eV (CQD‐CTA) and 0.44 eV (CQD‐ME) after surface reprogramming, which indicates that the doping character of the CQD ink is tuned from *n* type to *p* type. The calculated *E*_F_, VB, and conduction band (CB) are summarized in Figure 3c. The CB was deduced from the VB and the optical bandgap measured from UV–vis absorbance (Figure S3, Supporting Information). When the CQD‐ME inks were employed in devices (same structure shown in Figure 1b), the open‐circuit voltage (*V*_oc_) of devices increases with an increased amount of doping (Figure S4, Supporting Information). The ME doping results in downshifted *E*_F_ of CQD solids compared to control CQD solids (CQD‐PbI_2_)_._ This increases the built‐in potential and band bending at the ZnO/CQD interface,^[^ ^33^, ^34^, ^35^ ^]^ leading to an improvement of *V*_oc_ in devices. We note that CQD‐ME devices exhibit the highest PCE value with doping concentration of 1.6 × 10^−3 ^mmol mg^−1^ (Figure S5 and Table S2, Supporting Information), which we use as the optimal condition for ME doping. In contrast to CQD‐ME devices, CQD‐CTA devices exhibit a decrease of *V*_oc_ with an increased amount of CTA doping. Although CQD‐CTA exhibits a similar energy level compared to CQD‐ME, its higher degree of energetic disorder causes a decrease of *V*_oc_ in the devices.

**Figure 3:**
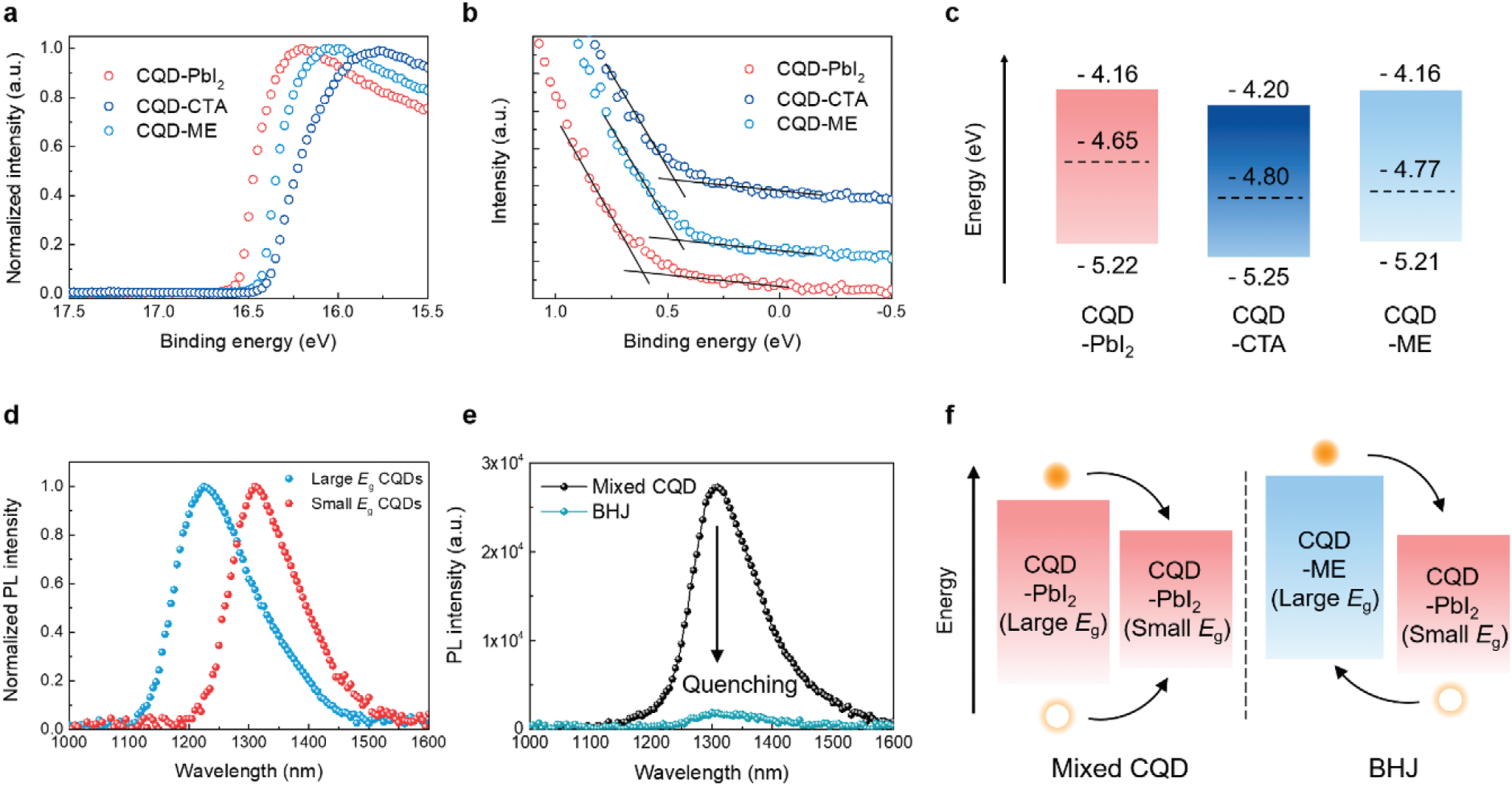
CQD‐PbI_2_ and CQD‐ME inks to fabricate CQD BHJ solids. UPS measurements of CQD inks for a) high‐binding‐energy cutoff and b) low‐binding‐energy cutoff. c) Energy level diagrams of full CQD devices deduced by UPS measurement. Optical bandgap measured by absorbance was used to estimate the conduction band edge position. d) Normalized PL intensity of large *E*_g_ CQDs (*E*_g_ = 1180 nm) and small *E*_g_ CQDs (*E*_g_ = 1250 nm) solids. e) PL intensity of mixed CQDs and CQD BHJ solid. The CQD BHJ film exhibits a strong quenching compared to the mixed CQDs. f) Schematic illustration of photoexcited carrier transfer in the CQD solids shown in (e).

We then pursued the fabrication of CQD BHJ solids by mixing multi‐*E*_g_ CQD inks—one is passivated with PbI_2_ (*n* type) and another is passivated with ME (*p* type). To verify enhanced carrier transport of CQD BHJ solids compared to mixed CQDs shown in Figure 1, we measured photoluminescence (PL). Figure 3d shows PL spectra of large *E*_g_ and small CQD *E*_g_ solids that is used for multi‐*E*_g_ CQD solids. Mixed CQDs consisting of both CQD‐PbI_2_ exhibit a strong PL intensity in the small *E*_g_ CQD population (Figure 3e). These form a type‐I heterojunction (Figure 3f): all carriers transfer from large *E*_g_ CQDs to small *E*_g_, not efficient to extract the carriers. On the other hand, CQD BHJ solids consisting of CQD‐PbI_2_ (small *E*_g_) and CQD‐ME (large *E*_g_) show a strong quenching of PL intensity, corresponding to the formation of a type‐II heterojunction, allowing efficient carrier extraction in the devices.

As an active layer in IR CQD solar cells, the CQD BHJ solids enables us to increase *J*_sc_ × FF of devices, with highest performance at an active layer thickness at a ≈715‐nm‐thick solid (**Figure** **4**a,b). To determine spectral charge extraction efficiency of the devices, we calculated a ratio of the EQE at the short circuit and −2 V.^[^ ^36^ ^]^ The CQD BHJ device shows near‐unity charge extraction efficiency with the 715‐nm‐thick solids, whereas the mixed CQD device has ≈80% charge extraction efficiency due to the limited extraction length. The inset in Figure 4c shows the internal quantum efficiency (IQE), which is in good agreement with charge extraction efficiency results. As a consequence, CQD BHJ devices exhibit EQE of >80% at the excitonic peak of both large *E*_g_ CQD (1180 nm) and small *E*_g_ CQD (1250 nm) as shown in Figure 4d. This results in a 30% enhancement of IR light harvesting beyond silicon bandgap (1100 nm). CQD BHJ devices exhibit reproducibly higher IR PCE (solar spectrum longer than 1100 nm) compared to mixed CQD devices with their optimal thickness of CQD solid (Figure 4e). A champion device records an IR PCE of 7.0%, corresponding to a *J*_sc_ of 5.50 mA cm^−2^ *V*_oc_ of 0.43 V, and an FF of 58% (Figure 4f), which adds an extra PCE of 1.37% to c‐Si solar cells.

**Figure 4:**
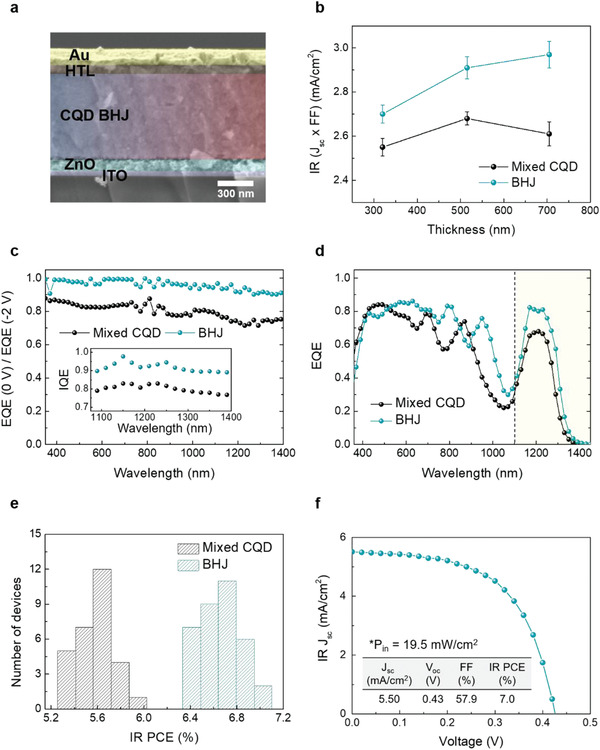
IR CQD BHJ devices. a) Scanning electron microscopy cross‐section image of the IR CQD BHJ device with 715‐nm‐thick CQD BHJ solid. b) Thickness‐dependent *J*_sc_ × FF values of IR CQD solar cells with solar spectrum beyond 1100 nm. Error bars represent the standard deviation of several devices. c) A ratio of the EQE at the short circuit and −2 V of CQD BHJ device and mixed CQD device when thickness of active layer is 715 nm. The inset shows IQE spectra of a corresponding device. d) EQE spectra of CQD BHJ device and mixed CQD device with their optimal thickness (≈500 nm for control and ≈700 nm for BHJ). Yellow region (longer than 1100 nm) highlights potential extra solar power beyond c‐Si solar cells. e) Histograms of device performance prepared with control CQD solids and CQD BHJ solids. The optimal thickness of CQD solids was used for devices. f) *J*–*V* characteristic of champion IR CQD BHJ device with 715‐nm‐thick active layer.

In summary, we report a CQD BHJ solid that enhances the charge carrier extraction for IR light harvesting. This is achieved by a colloid‐phase doping strategy, specific for IR CQDs and that enables to tune their doping character and *E*_F_ while preserving their homogeneity. It enables the IR CQD BHJ architecture which exhibits charge extraction efficiency of >90% in all active wavelengths (350–1400 nm) with 700‐nm‐thick CQD solids. As a result, the CQD BHJ device shows EQE of >80% and enhanced PCE in the spectral regime beyond the response of silicon.

## Experimental Section

##### Synthesis of IR Colloidal Quantum Dots

Oleic‐acid‐capped CQDs were synthesized using a method described in the literature.^[^ ^10^ ^]^ Specifically, for a synthesis of CQDs with an exciton peak at 1150 nm, 1.35 g of PbO, 4.5 mL of oleic acid, and 15 mL of 1‐octadecene were mixed in three‐neck flask and degassed at 100 °C for 2 h to form transparent lead‐oleate solution. Then, the flask filled with N_2_ and temperature increased to 115 °C. The 210 µL of bis(trimethylsilyl)sulfide was dissolved in 8 mL of 1‐octadecene in the glove box and swiftly injected into the mixed solution. The color of the solution changed to brown rapidly and slowly cooled down to room temperature. The as‐synthesized solution was moved to the glove box and precipitated by adding acetone. This washing step was repeated three times and final CQD powder was dissolved in octane with concentration of 50 mg mL^−1^ and stored in N_2_‐filled glove box.

##### Synthesis of IR Colloidal Quantum Dot Inks

All processes were done in ambient air condition. To form *n*‐type CQD inks, 20 mL of CQD solution dissolved in octane (7 mg mL^−1^) was added to 20 mL of precursor solution (lead iodide 0.1 m and lead bromide 0.02 m, and NaAc 0.055 m in DMF).^[^ ^24^ ^]^ Then the solution was mixed vigorously for 5 min until CQDs were transferred to the DMF phase. The octane was removed and the DMF solution was washed with octane three times. The DMF solution was precipitated by adding toluene and centrifuging the solution. The yellow supernatant was discarded and the precipitated CQDs were dried in vacuum. The CQD powder was redispersed in mixture of butylamine:DMF (4:1 volume ratio) solution. To from *p*‐type CQD inks, a 100 µL of ME solution (0.18 mm in DMF) was slowly introduced to the above CQD DMF solution before precipitation. The other processes were same with the *n*‐type CQD inks.

##### Fabrication of IR CQD Solar Cells

The ZnO nanoparticles were synthesized using a published method.^[^ ^3^ ^]^ The ZnO nanoparticles were spin cast on an ITO substrate at 5000 rpm for 30 s. This process was repeated once more. Then the mixed CQD inks (mixture of the *n*‐type CQD inks and *p*‐type CQD inks with 1:1 weight ratio) were spin cast onto the ZnO/ITO substrate at 800 rpm for 30 s. The dynamic spin‐coating method was used to achieve uniform film: the CQD solution dropped while the substrate was spinning at 800 rpm. Various concentrations of the mixed CQD inks (250–380 mg mL^−1^) was used to change a thickness of the CQD film. Then, PbS CQD layer treated with 1,2‐ethandithiol (EDT) was deposited as a hole transport layer. Oleic‐acid‐capped PbS CQDs (*E*_g_ = 1.3 eV) were spin cast, and then soaked with 0.01 vol% EDT solution in ethyl acetate for 30 s, followed by three repetitions of washing using ethyl acetate. This process was repeated once more. Finally, 120 nm of Au was deposited via e‐beam evaporation as the top electrode.

##### Solar Cell Measurements

The active area (0.049 cm^2^) was determined by the aperture placed between the devices and the AM1.5 solar simulator (Sciencetech class A). Current–voltage characteristics were measured with the aid of a Keithley 2400 source measuring unit under simulated AM1.5 illumination. Devices were tested under a continuous nitrogen flow. The *I*–*V* curves were scanned from −0.70 to +0.1 V at 0.02 V interval steps without wait time between voltage steps. The spectral mismatch was calibrated using a reference solar cell (Newport). A 1100 nm long‐pass filter was used to measure IR characteristics. Input power of 19.5 mW cm^−2^ was used to calculate IR PCE of devices. EQE spectra were taken by subjecting the solar cells to chopped (220 Hz) monochromatic illumination (400 W Xe lamp passing through a monochromator and appropriate cutoff filters). Newport 818‐UV and Newport 838‐IR photodetectors were used to calibrate the output power. The response of the cell was measured with a Lakeshore preamplifier feeding into a Stanford Research 830 lock‐in amplifier at short‐circuit conditions. IQE spectra were determined by EQE/[1 − *R*(*hv*)], where *R*(*hv*) is the reflectance at a photon energy of *hv*. *R* was derived from *A* = 1 − *R*, where *A* is double‐pass absorption mode of the CQD devices, which were fully covered with Au metal electrode.

##### Other Characterization

Photoluminescence measurements were carried out using a Horiba Fluorolog Time Correlated Single Photon Counting system equipped with UV–vis–NIR photomultiplier tube detectors, dual grating spectrometers, and a monochromatized xenon lamp excitation source. The PL lifetime data was recorded on using a time‐correlated single‐photon counting system (Horiba). Optical absorption measurements were carried out in a Lambda 950 UV–vis–IR spectrophotometer. Absorption of films was measured by using integral sphere. XPS analysis was performed using a Thermo Scientific K‐Alpha XPS system (300 µm spot size, 75 eV pass energy, and 0.05 eV energy steps). The XPS spectra were calibrated to the C 1s peak at a binding energy of 284.8 eV.

##### Computational Method

Electronic structure calculations were performed in the framework of DFT^[^ ^37^ ^]^ and Perdew–Burke–Ernzerhof generalized gradient approximation^[^ ^38^ ^]^ (GGA‐PBE) for the exchange‐correlation functional. Van der Waals correction was considered for all calculations at a PBE + D3 level.^[^ ^39^ ^]^ Total energies of all calculations were obtained using the Vienna ab initio simulation program with projector augmented‐wave potentials.^[^ ^40^, ^41^, ^42^ ^]^ The (100) PbS slab was modelled as 1 × 2 × 3 supercell spaced by 18.3 Å. For reciprocal space integration, 4 × 2 × 1 Monkhorst–Pack grid^[^ ^43^ ^]^ was used. The cutoff energy for a plane wave expansion was set at ≈500 eV. The bottom layer of the slab was fixed to their bulk positions during relaxation, while the rest of the layers were relaxed. The atomic positions were optimized with the convergence threshold 10^−7 ^eV for energy and 0.01 eV Å^−1^ for force, respectively.

## Conflict of Interest

The authors declare no conflict of interest.

## Supporting information

Supporting Information
