# Supplementary material for: Colloidal Quantum Dot Bulk Heterojunction Solids with Near‐Unity Charge Extraction Efficiency
Source: Adv Sci (Weinh). 2020 Jun 17;7(15):2000894. doi: 10.1002/advs.202000894 (PMC7404161; doi:10.1002/advs.202000894)
Supplement: Supplementary file 1 — Supporting Information [file ADVS-7-2000894-s001.pdf]

© 2020 Wiley-VCH GmbH

ADVANCED SCIENCE Open Access

Supporting Information

for *Adv. Sci.*, DOI: 10.1002/advs.202000894

Colloidal Quantum Dot Bulk Heterojunction Solids with Near-Unity Charge Extraction Efficiency

*Min-Jae Choi, Se-Woong Baek, Seungjin Lee, Margherita Biondi, Chao Zheng, Petar Todorovic, Peicheng Li, Sjoerd Hoogland, Zheng-Hong Lu, F. Pelayo García de Arquer, and Edward H. Sargent**

Copyright WILEY-VCH Verlag GmbH & Co. KGaA, 69469 Weinheim, Germany, 2020.

Supporting Information

Colloidal Quantum Dot Bulk Heterojunction Solids with Near-Unity Charge Extraction Efficiency

*Min-Jae Choi, Se-Woong Baek, Seungjin Lee, Margherita Biondi, Chao Zheng, Petar Todorovic, Peicheng Li, Sjoerd Hoogland, Zheng-Hong Lu, F. Pelayo García de Arquer, and Edward H. Sargent**

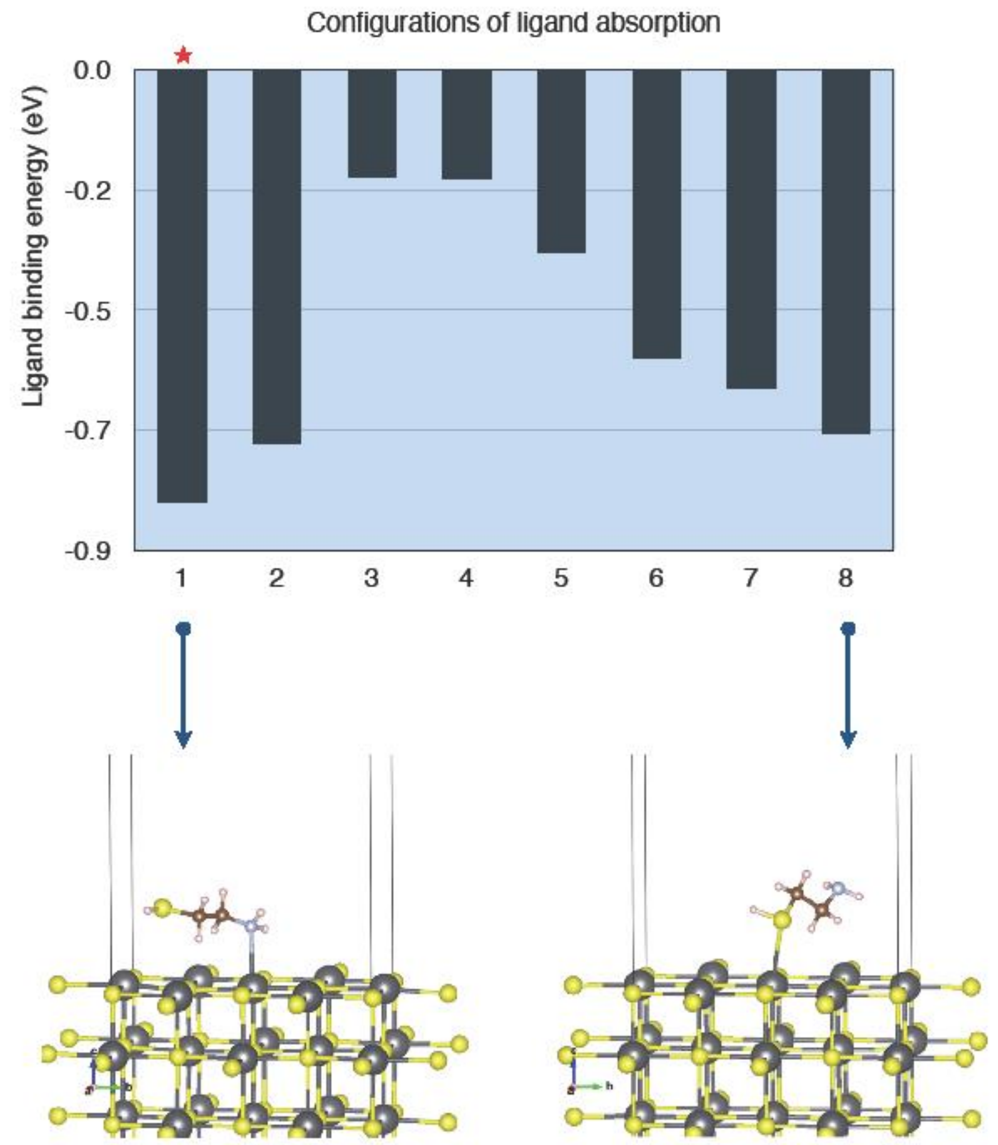

Figure S1. DFT calculation of absorption energy when CTA ligand binds with PbS (100) facet . The black dot represents Pb atoms, yellow dot represents S atoms, brown dot represents C atoms, and light-blue dot represents N atoms. We designed 8 initial CTA absorption configurations. The stable configuration is estimated via calculating binding energy E_b,

$$E_b = E_{surface+L} - E_L - E_{surface}$$

In which, $E_{surface+L}$ is the total energy of the (100) PbS surface passivated by CTA and E_L is the total energy of CTA. The total energy of the clean (100) PbS surface is represented by $E_{surface}$. The most negative binding energy represents the stable configuration. From the calculation, the first configuration has the lowest E_b which clearly indicates the amine

functional group bonding with one surface Pb atom from (100) PbS and the thiol group obliquely points to the vacuum space. This observation suggests the preference of amine group from CTA binds with (100) PbS surface.

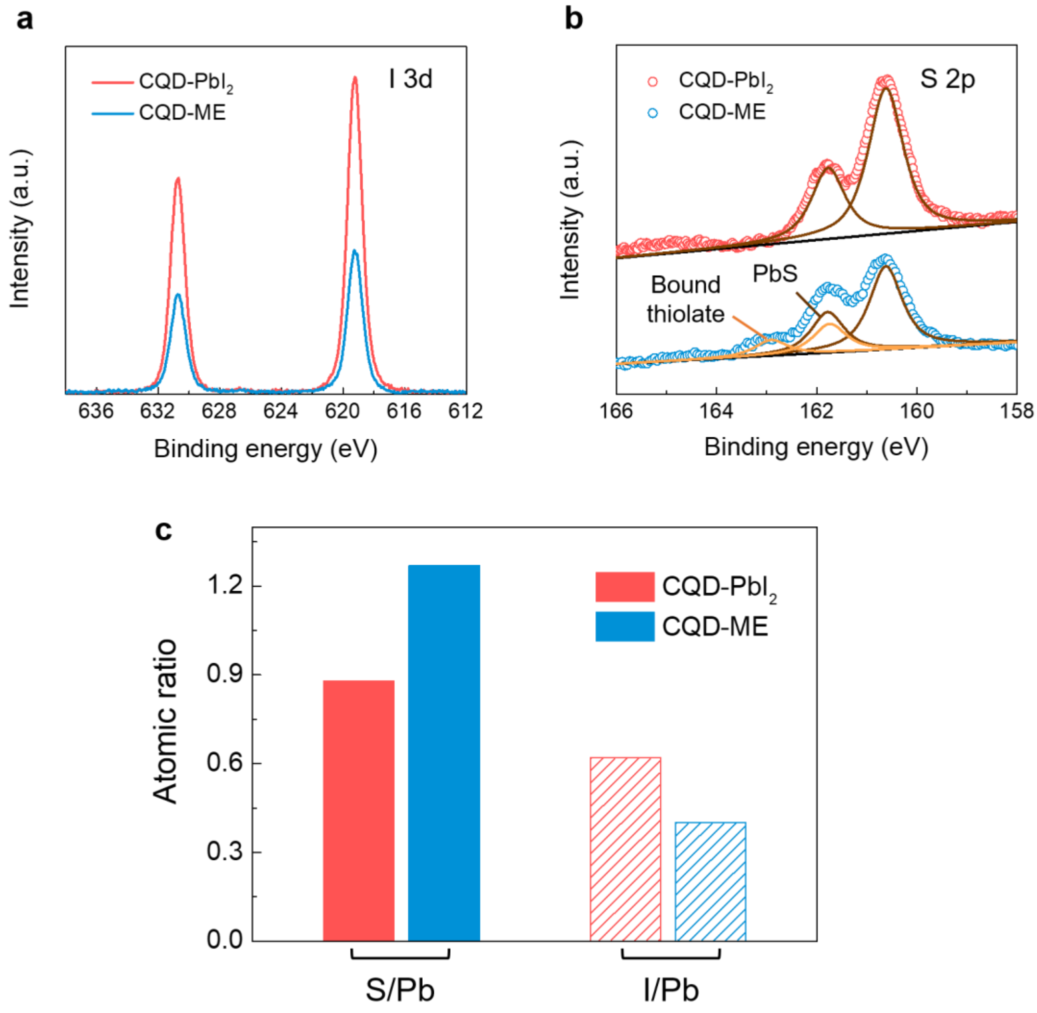

Figure S2. a-b) XPS I 3d spectra (a) and S 2p spectra (b) of CQD-PbI_2 and CQD-ME CQD inks. (c) Sulfur to lead (S/Pb) and iodine to lead (I/Pb) atomic ratio of CQD-PbI_2 and CQD-ME CQD inks.

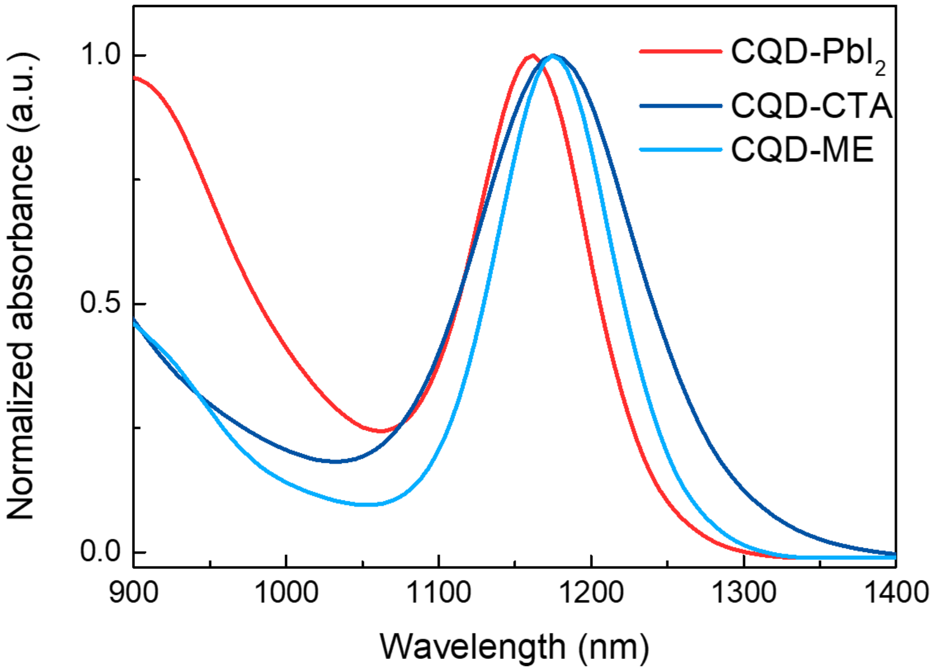

Figure S3. Normalized absorption spectra of CQD solids after ligand exchange.

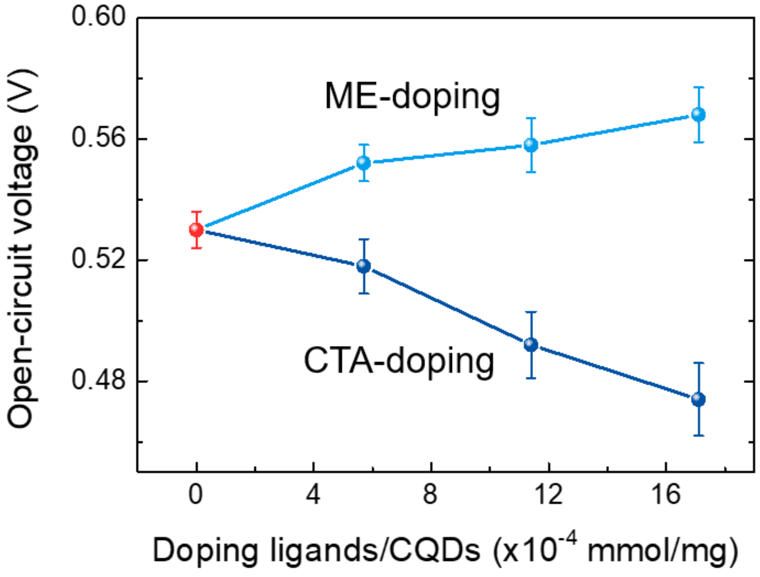

Figure S4. Open-circuit voltage (V_{oc}) of CQD solar cells prepared by CQD-ME and CQD-CTA inks under AM1.5 illumination. An increased amount of ME doping increases V_{oc}, whereas an increased amount of CTA decreases V_{oc}.

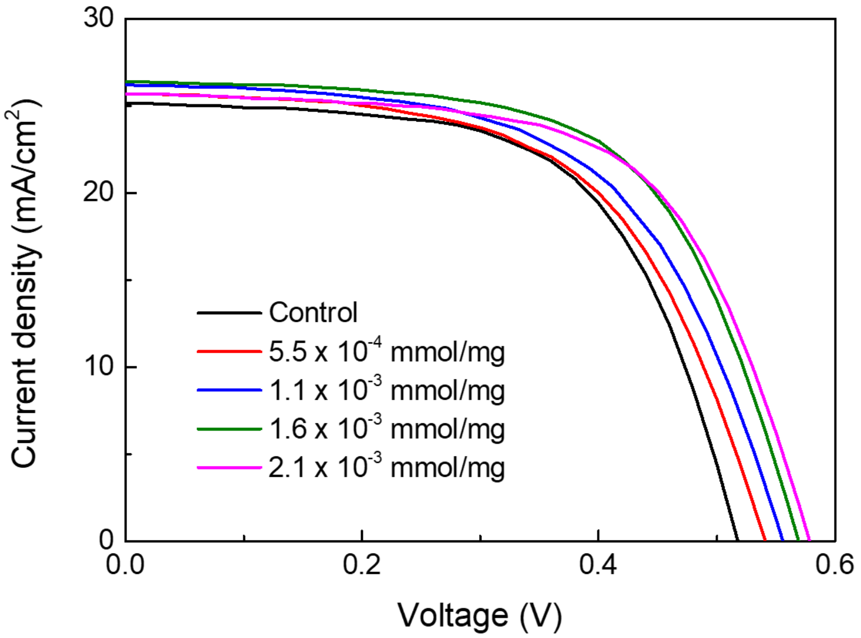

Figure S5. J-V characteristics of CQD solar cells prepared by CQD-ME inks with different doping concentration. Thickness of active layer was ~300 nm and the measurements were done under AM1.5 illumination.

Table S1. Atomic ratio of sulfur, iodide, and nitrogen (to lead) of PbS CQD inks before and after CTA-reprogramming.

Sample	S/Pb	I/Pb	N/Pb
Before CTA-reprogramming	0.88	0.62	0
After CTA-reprogramming	1.29	0.45	0.14

Table S2. Device performance of CQD solar cells in Figure S4.

Doping concentration	V_{oc} (V)	J_{sc} (mA/cm^2)	FF (%)	PCE (%)
Control	0.515	25.2	58.8	7.6
5.5 x 10^{-4} mmol/mg	0.543	25.7	58.9	8.2
1.1 x 10^{-3} mmol/mg	0.555	26.0	59.3	8.6
1.6 x 10^{-3} mmol/mg	0.568	26.2	62.1	9.2
2.1 x 10^{-3} mmol/mg	0.577	25.6	60.3	8.9
